# Structure‐Crack Detection and Digital Twin Demonstration Based on Triboelectric Nanogenerator for Intelligent Maintenance

**DOI:** 10.1002/advs.202302443

**Published:** 2023-07-06

**Authors:** Chuanfu Xin, Zifeng Xu, Xie Xie, Hengyu Guo, Yan Peng, Zhongjie Li, Lilan Liu, Shaorong Xie

**Affiliations:** ^1^ School of Mechatronic Engineering and Automation Shanghai University Shanghai 200444 P. R. China; ^2^ Shanghai Key Laboratory of Intelligent Manufacturing and Robotics Shanghai University Shanghai 200444 P. R. China; ^3^ Department of Applied Physics Chongqing University Chongqing 400044 P. R. China; ^4^ Institute of Artificial Intelligence Shanghai University Shanghai 200444 P. R. China; ^5^ Engineering Research Center of Unmanned Intelligent Marine Equipment Shanghai University Shanghai 200444 P. R. China; ^6^ School of Computer Engineering and Science Shanghai University Shanghai 200444 P. R. China

**Keywords:** convolutional neural networks, defect detection, digital twin, Gramian angular field, triboelectric nanogenerators

## Abstract

The accomplishment of condition monitoring and intelligent maintenance for cantilever structure‐based energy harvesting devices remains a challenge. Here, to tackle the problems, a novel cantilever‐structure freestanding triboelectric nanogenerator (CSF‐TENG) is proposed, which can capture ambient energy or transmit sensory information. First, with and without a crack in cantilevers, the simulations are carried out. According to simulation results, the maximum change ratios of natural frequency and amplitude are 1.1% and 2.2%, causing difficulties in identifying defects by these variations. Thus, based on Gramian angular field and convolutional neural network, a defect detection model is established to achieve the condition monitoring of the CSF‐TENG, and the experimental result manifests that the accuracy of the model is 99.2%. Besides, the relation between the deflection of cantilevers and the output voltages of the CSF‐TENG is first built, and then the defect identification digital twin system is successfully created. Consequently, the system is capable of duplicating the operation of the CSF‐TENG in a real environment, and displaying defect recognition results, so the intelligent maintenance of the CSF‐TENG can be realized.

## Graphical abstract

A novel cantilever‐structure triboelectric nanogenerator (CSF‐TENG) is proposed to achieve the condition monitoring and intelligent maintenance of energy harvesting devices. Based on Gramian angular field and convolutional neural network, the defect detection model with an accuracy of 99.2% is established. Besides, the digital twin system is created, which can duplicate the operation of the CSF&TENG and display defect recognition results.

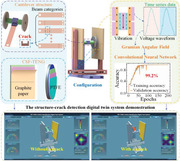

## 1 Introduction

With the development of 5G and information technology, society has gradually entered the era of Internet of Things, big data, and artificial intelligence. To realize the interconnections of people, machines, and things, the number of sensors shows an explosive increase, which possess the characteristics of scattered distribution, low power consumption, and so on. However, aiming at supplying power for massive sensors, traditional energy sources (such as chemical batteries with high replacement costs) can no longer meet their demands. To support the operation of sensor networks, how to efficiently and stably harvest ambient energy has become a hot research topic. Xin et al. investigated the output performance of triboelectric nanogenerators embedded interlayers, and the experimental results manifest that the output voltage has increased by five times.^[^ ^1^ ^]^ Li et al. proposed a high‐efficiency electromagnetic generator with abrupt magnetic flux density change, which can charge a 20 mF capacitor from 0 to 30 V within 50 s.^[^ ^2^ ^]^ Li et al. developed a piezo stack‐based frequency‐converted generator, which can yield an average power of 0.93 mW.^[^ ^3^ ^]^ Kang et al. designed a pyroelectric generator (PEG) to scavenge the ambient heat energy, and the PEG can light up 15 LEDs.^[^ ^4^ ^]^

With the advantages of effective scavenging vibration energy, easy coupling with the environment and fabrication, the cantilever structure‐based energy harvesting devices are commonly used to capture ambient energy, such as wind,^[^ ^5^ ^]^ mechanical vibration,^[^ ^6^ ^]^ and wave.^[^ ^7^ ^]^ Due to the installation location, installation geographical environment, etc., the fatigue strength and performance of components continuously decline with the increase of operating time, causing cracks in the overstress area of cantilevers. To ensure that the cantilever structure‐based energy harvesting devices can capture ambient energy to supply power for sensor networks continuously, detecting damage is essential. Elshamy et al. utilized the first three modes of vibration to detect cracks in a cantilever beam, and the results manifest that the identification error is small.^[^ ^8^ ^]^ Khiem et al. developed a procedure for identifying a single crack by using the modal charge of piezoelectric sensors.^[^ ^9^ ^]^ Compared to the natural frequency, the modal charge is more efficient. Zhang et al. presented a method of diagnosing multi‐cracks in a cantilever beam, and the results show that the locations and depths of cracks can be accurately identified.^[^ ^10^ ^]^ Zhao et al. proposed a crack identification method for cantilever structures by using piezoelectric materials, which can bring about a 25% change in the natural frequencies of cantilever beams, enabling more effective crack detection.^[^ ^11^ ^]^

Although many crack detection methods for beam‐type structures have been developed, most of them use a sensor relying on external power to collect signals, causing obstacles in detecting damage of energy harvesting devices on a large scale. Furthermore, because of the disadvantages of complex installation, brittleness, and easily broken under impact loads, piezoelectric materials are not suitable for curved structures with large amplitude variations. Accordingly, it is essential to develop energy harvesting devices with the characteristics of self‐generated electrical signals, high bending resistance, and easy integration. Noticeably, TENG is suitable, because TENG owns the advantages of various materials, flexible, lightweight, easy fabrication, etc.^[^ ^12^ ^]^ What's even more significant is that TENG can produce self‐generated electrical signals in response to contact separation, contact sliding, and deformation, which simplifies the design of signal acquisition circuits considerably. Thus, TENG has been investigated to transmit sensory information. Mehamud et al. designed a TENG‐based vibration sensor with a wide range of frequency detection (0–1200 Hz) to monitor machine conditions, and the results show that it can provide the same function as commercial vibration sensors.^[^ ^13^ ^]^ Hu et al. developed a TENG multi‐span rotor system to monitor conditions of its own, and by ensemble learning, the diagnostic accuracy can reach 95.76%.^[^ ^14^ ^]^ Based on the triboelectric effect, Han et al. achieved the self‐powered fault diagnosis of rolling bearing, and the results manifest that the classification accuracy can reach 92%.^[^ ^15^ ^]^ Xie et al. proposed a triboelectric planetary gear sensor to monitor gear faults of the planetary reducer, and based on convolutional neural network (CNN), the classification accuracy can achieve 100%.^[^ ^16^ ^]^

Additionally, aiming at realizing intelligent maintenance of devices, digital twin provides an effective strategy. Digital twin is regarded as the best technology to achieve the integration of the physical world and digital space, which possesses the characteristics of virtual‐real mapping, data‐driven, and so on.^[^ ^17^ ^]^ By digital twin, maintenance personnel can quickly pinpoint the location of device failures without the necessity of on‐site physical inspections, thereby improving maintenance efficiency. Combining TENG and digital twin, Jin et al. built a soft‐robotic gripper system, which displays virtual robotic manipulation.^[^ ^18^ ^]^ Based on Jin's research, Sun et al. further investigated the potential of the soft‐robotic gripper system in digital‐twin‐based virtual shop applications.^[^ ^19^ ^]^ Subsequently, Zhang et al. reported a 3D scanning system based on triboelectric sensors for accurately collecting point cloud data and digital twin applications.^[^ ^20^ ^]^ Yang et al. developed a digital‐twin smart home, which can duplicate the status of walking trajectory and dynamic activities to digital space.^[^ ^21^ ^]^

Although TENG has been utilized in the field of fault diagnosis, few studies were reported in recognizing faults and visualizing them to achieve intelligent maintenance of devices based on triboelectric sensors and digital twin. In addition, it remains a challenge in utilizing triboelectric signals to directly drive 3D models in virtual space for realizing virtual‐real mapping. Herein, a cantilever structure‐based energy harvesting device was designed based on TENG, which is utilized as a self‐powered sensor to detect damage of the cantilever‐structure freestanding triboelectric nanogenerator (CSF‐TENG) via Gramian angular field (GAF) and CNN. The main contributions are as follows: (1) the defect detection model was established via GAF and CNN, realizing the condition monitoring of the CSF‐TENG; (2) to achieve intelligent maintenance of the device, the defect identification digital twin system of the CSF‐TENG was built; (3) the relation between the deflection of cantilevers and the output voltage of the CSF‐TENG was established; (4) a novel CSF‐TENG was designed; (5) the simulations of cantilevers with cracks at different positions were performed to acquire the changes of natural frequency and amplitude.

## 2 Results and Discussion

### 2.1 Configuration and Working Mechanism of the CSF‐TENG

**Figure 1:**
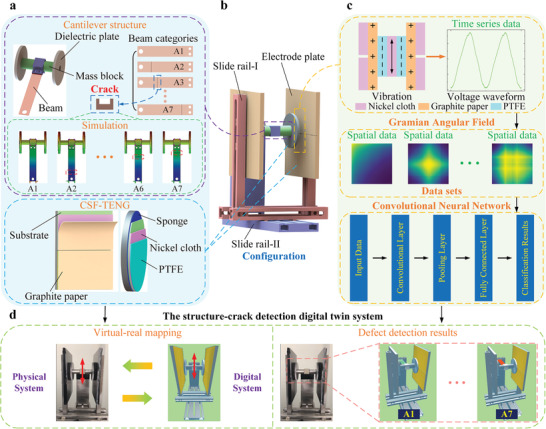
Configuration of the CSF‐TENG for structure‐crack detection and digital twin demonstration. a) The components of the CSF‐TENG. b) The 3D model of the CSF‐TENG. c) The defect detection of the CSF‐TENG. d) The defect identification digital twin system of the CSF‐TENG.

In this paper, the CSF‐TENG device is a symmetrical configuration (as shown in **Figure** **1**b), and the mechanical model is illustrated in Figure S1, Supporting Information. As described in Figure 1a, the device is mainly composed of electrode plates, dielectric plates, mass blocks, cantilevers, and slides. The cantilever with a size of 64 × 12 × 0.4 mm is made of 65Mn spring steel. Similarly, the dielectric plates with a diameter of 40 mm are made of acrylic plate material. The rest of the components are made of 45 steel, and their specifications are depicted in Figure S2, Supporting Information. To make the preload between the positive and negative triboelectric layers adjustable, and avoid the influence of the contact force between two triboelectric layers on the vibration behavior of the beam itself, slide rail‐1 was designed to move back and forth on slide rail‐2. Furthermore, the sponge is utilized as a buffer layer to increase the contact area of positive and negative triboelectric layers, which has been proved in previous studies,^[^ ^22^ ^]^ and the polyimide (PI) is used as a substrate to prevent the electrical output of the CSF‐TENG from being affected.

Under external excitation, the cantilever will drive the dielectric plate to swing up and down. Due to the difference in the electron affinities of positive and negative triboelectric layers, the surface of the polytetrafluoroethylene (PTFE) triboelectric layer accumulates negative charge, while the surface of the graphite paper (Gp) triboelectric layer gathers an equivalent quantity of positive charge. Based on contact electrification and electrostatic induction, the upper and lower electrodes will produce equal and different polar charge. As illustrated in Figure S3a, Supporting Information, when external excitation is not applied to the cantilever, the upper and lower electrodes keep an electrostatic balance, exhibiting no output current. Under external excitation, the cantilever swings upward, and the electrostatic balance between the upper and lower electrodes is broken. The upper electrode will accumulate positive charge, and the lower electrode will gather negative charge. Thus, currents flow from the lower electrode to the upper electrode through an external load circuit. Next, the cantilever moves downward. The cantilever first returns to the initial position, and then moves downward continuously. Similarly, the upper electrode will accumulate negative charge, and the lower electrode will gather positive charge. Therefore, currents flow from the upper electrode to the lower electrode through an external load circuit. Finally, the cantilever returns to the initial position, and the upper and lower electrodes again restore electrostatic balance. In addition, the simulation results of the CSF‐TENG in COMSOL 5.6 are displayed in Figure S4, Supporting Information, and the experimental platform and testing circuit are shown in Figure S5, Supporting Information.

Under the ambient periodic excitation, the CSF‐TENG is used to convert the vibration energy into electrical energy or transmit sensory information. Nonetheless, the CSF‐TENG is prone to crack damage in long‐term operation, especially in the over‐stress area. Under cantilevers with and without a crack, the maximum change ratios of natural frequency and amplitude are slight according to simulation results (as shown in Figure 1a), which makes it difficult to identify defects based solely on these variations. To capture environmental energy continuously, it is necessary to monitor the conditions of the CSF‐TENG with some high‐performance methods. Here, based on triboelectric sensory information, GAF, and CNN, the defect detection model of the CSF‐TENG is established, as illustrated in Figure 1c. In addition, to achieve intelligent maintenance of the device, the defect identification digital twin system of the CSF‐TENG is built (as described in Figure 1d), which can duplicate the operation of the CSF‐TENG in a real environment and display defect recognition results. Digital twin can link the physical system to the cyber world of computation (referring to Figure S3b, Supporting Information) through advanced communication technologies, so that the maintenance efficiency of energy harvesting devices can be significantly improved.

### 2.2 Simulations of the CSF‐TENG with Cracks at Different Positions

**Figure 2:**
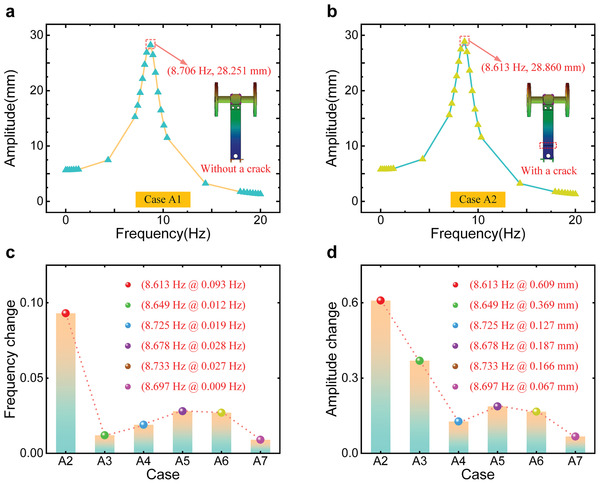
The simulation results of cantilevers under different cases. The simulation results of cantilevers under a) Case A1 and b) Case A2. c) The frequency changes of cantilevers under different cases. d) The amplitude changes of cantilevers under different cases.

In the long‐term operation of devices, damage is inevitable, such as wear, corrosion, and fracture. Effective monitoring methods can detect device damage in the early stages, so maintenance can be done timely. For the crack damage of cantilevers, some researchers have proposed to utilize the changes of natural frequency or amplitude to detect defection, for example, a frequency‐based methodology for cantilever crack detection,^[^ ^11^ ^]^ a method for crack identification in cantilever structure by utilizing the structure's mode shapes,^[^ ^23^ ^]^ and spotting cantilever's crack based on the first three natural frequencies in fast Fourier transform.^[^ ^24^ ^]^ Compared with non‐defective cantilevers, the natural frequency and amplitude changes of cantilevers with cracks at different positions are diverse. To explore the natural frequency and amplitude variations of cantilevers in different cases, we performed simulations by using Solidworks 2018. The simulation conditions are set as follows: the excitation frequency range is set to 0–20 Hz, the force applied on the end frame surface is 5 N (uniformly distributed), and the modal damping is set to 0.1. The simulation results are shown in **Figure** **2**.

According to the different distances between the defect position and the fixed end of cantilevers, we set six defect locations, that is, Case A2, Case A3, Case A4, Case A5, Case A6, and Case A7. When the cantilever is defect‐free (Case A1), the simulation results are shown in Figure 2a. With the increase of excitation frequency, the amplitude of the cantilever rises first and then decreases, which can reach the maximum under the excitation of natural frequency. When the excitation frequency is 8.706 Hz, the amplitude of the cantilever without a crack reaches a maximum of 28.251 mm. Next, the cantilever's amplitude was measured under Case A2. As depicted in Figure 2b, the change trend of the cantilever's amplitude is similar to that of the cantilever without a crack. Under the excitation frequency of 8.613 Hz, the amplitude is 28.860 mm. Compared with Case A1 and Case A2, the natural frequency of the cantilever in Case A2 decreases slightly, but the amplitude of the cantilever increases marginally. Then, we performed simulations under Case A3, Case A4, Case A5, Case A6, and Case A7, and the results are displayed in Figure S6, Supporting Information.

To compare the natural frequency and amplitude changes of cantilevers with cracks at different positions, Equations (1) and (2) are used to calculate the variation ratios of the natural frequency and amplitude, that is, Case (A1, A2), Case (A1, A3), Case (A1, A4), Case (A1, A5), Case (A1, A6), and Case (A1, A7).

(1) $$f_{\mathrm{VR}}=\left|f_{AN}-f_{A1}\right|/f_{A1}\;N=2,3,\text{…},7$$

(2) $$a_{\mathrm{VR}}=\left|a_{AN}-a_{A1}\right|/a_{A1}\;N=2,3,\text{…},7$$

where *f*_VR_ and *a*_VR_ are the variation ratios of the natural frequency and amplitude, and *f*_A*N*_ and *a*_A*N*_ are the natural frequency and amplitude, respectively.

As shown in Figure 2c, under cantilevers with cracks at different positions, the variation ratios of natural frequency are 1.1%, 0.1%, 0.2%, 0.3%, 0.3%, and 0.1%, respectively. Noticeably, the change of natural frequency in Case A2 is the largest, which is because the crack is closest to the fixed end of cantilevers, resulting in the greatest influence on its stiffness. As illustrated in Figure 2d, under cantilevers with cracks at different positions, the variation ratios of amplitude are 2.2%, 1.3%, 0.4%, 0.7%, 0.6%, and 0.2%, respectively. Similarly, the maximum amplitude variation also occurs in Case A2. According to the above simulation results, the maximum change ratios in natural frequency and amplitude are 1.1% and 2.2%, respectively. The variations are slight, which will make it difficult to identify crack defects based on the natural frequency and amplitude changes, so it is necessary to develop other crack defect identification methods of the CSF‐TENG.

### 2.3 The Crack Defect Detection of the CSF‐TENG

To ensure that the CSF‐TENG can continuously convert the environmental vibration energy into electrical energy for powering sensing networks, effective crack defect identification methods are essential. According to the above simulation results, the natural frequency and amplitude of cantilevers with cracks at different positions change slightly, so identifying cantilever crack defects based on these variations is not applicable. According to experimental results, when the crack occurs at different positions, the electrical signal waveforms of the CSF‐TENG are distinct under the same excitation conditions. Here, based on GAF, CNN, and electrical signal waveforms, a method of identifying cantilever crack defects is proposed.

**Figure 3:**
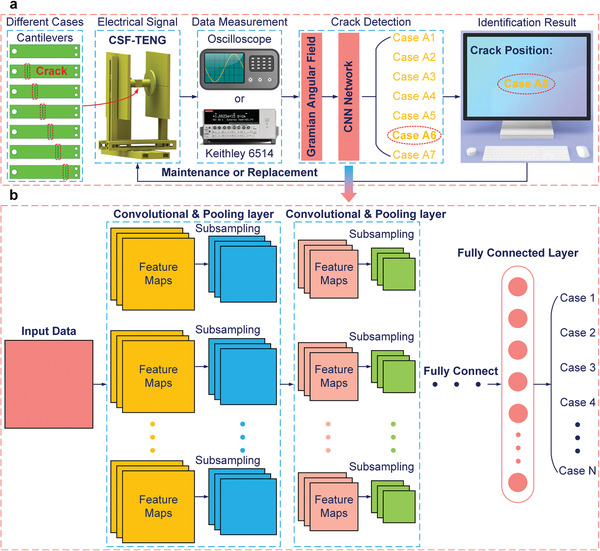
The crack defect detection of the CSF‐TENG. a) The flow diagram of identifying cantilever crack defects. b) The CNN model structure diagram.

**Figure** **3**a exhibits the flow diagram of identifying cantilever crack defects based on GAF and CNN. According to the different distances from cracks to the fixed end of cantilevers, six defect positions were set, and the length, width, and depth of each crack are 12 × 0.4 × 0.2 mm. Under the excitation acceleration of 0.4 g and the excitation frequency of 8 Hz, different cantilevers were tested in sequence, and the electrical signal waveforms of the CSF‐TENG were collected by an oscilloscope or an electrometer. The obtained electrical signal waveforms are first preprocessed by the GAF method, and then divided into training sets, validation sets, and test sets according to a certain proportion (7:2:1) to establish the crack defect detection model of the CSF‐TENG based on CNN. Finally, the identification results are displayed on the PC to realize the intelligent maintenance of the CSF‐TENG. Figure 3b is the structure diagram of CNN, which consists of convolutional layers, pooling layers, and fully connected layers.

To build the crack defect detection model, the electrical signal samples of the CSF‐TENG are first collected by an oscilloscope under different cases. When cantilevers with and without a crack are used, the number of signal feature points is the key to identify them. Thus, to capture as many signal features as possible, the experimental conditions are set as follows: (1) to collect more details of the voltage waveform, the excitation frequency is set to 8 Hz; (2) the sampling time is set to 40 s; (3) the sampling rate is set to 25 kS s^−1^. In addition, the electrical signal data collected within 1 s is used as eight samples, so the data dimension of each sample is about 3125. Although the number of signal features can be ensured, if the original data is directly used to establish the defect detection model, the training efficiency of the model will be very low. To reduce data dimension and retain feature information simultaneously, some data dimensionality reduction algorithms have been proposed, such as wavelet algorithm,^[^ ^25^ ^]^ principal component analysis,^[^ ^26^ ^]^ independent component analysis,^[^ ^27^ ^]^ factor analysis,^[^ ^28^ ^]^ and linear discriminant analysis.^[^ ^29^ ^]^ However, the performance of the model depends heavily on the setting parameters of the data dimensionality reduction algorithms. Furthermore, the parameter optimization of the data dimensionality reduction algorithms is complicated. Hence, to improve the training efficiency and accuracy of the defect detection model, the GAF method is used to preprocess the sample data.

GAF is a method to convert 1D time series signal data into 2D image data, which possesses the advantages of retaining the time series characteristic of data and containing the complete information of original time series signals. The electrical signals of the CSF‐TENG are typical 1D time series data. The horizontal coordinate of the sample data is the time value, and the vertical coordinate is the voltage value corresponding to each sampling point. Hence, the GAF method is used to preprocess data, and the process of converting 1D time series signals into 2D image data is shown in Note S1, Supporting Information.

**Figure 4:**
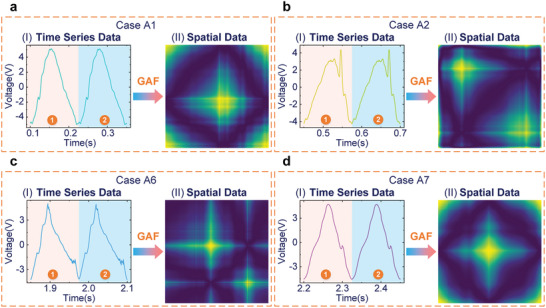
The results of converting time series data into spatial data. The converting results are under a) Case A1, b) Case A2, c) Case A6, and d) Case A7.

By using Gramian angular summation fields, 1D time series data (The two identical time series samples under different cases are displayed in **Figure** **4**a–d.) of the CSF‐TENG is converted into 2D spatial data, as shown in Figure 4 and Figure S7, Supporting Information. According to the measured results, the electrical signal waveforms of the CSF‐TENG are diverse under different cases, which is beneficial to identify crack defects in cantilevers. Compared with the transformation results of Case A1 and Case A2, the 2D feature diagrams can be easily distinguished. This is because the crack is close to the fixed end of the cantilever in Case A2, which has a great influence on the cantilever's vibration mode. However, comparing the transformation results of Case A1 and Case A7, the 2D feature maps are indistinguishable. This is because the crack has a large distance to the fixed end of the cantilever in Case A7, so a small impact on the vibration mode of the cantilever is generated, which is consistent with the simulation results of cantilevers.

Then, based on 2D spatial data and CNN, the defect detection model of the CSF‐TENG was established. CNN is a feedforward neural network inspired by natural cognitive mechanisms, and the manual construction of complex mathematical models can be avoided by establishing a nonlinear mapping between the input sample and output target. As shown in Figure 3, CNN is mainly composed of the input layer, hidden layer (containing multiple convolutional layers, pooling layers, fully connected layers, and activation function, as shown in Note S2, Supporting Information), and output layer. The resolution of image features is first reduced by the alternating operation of the convolution layer and the pooling layer, and then they are mapped to the fully connected layer by activation function to obtain sample labels.

**Figure 5:**
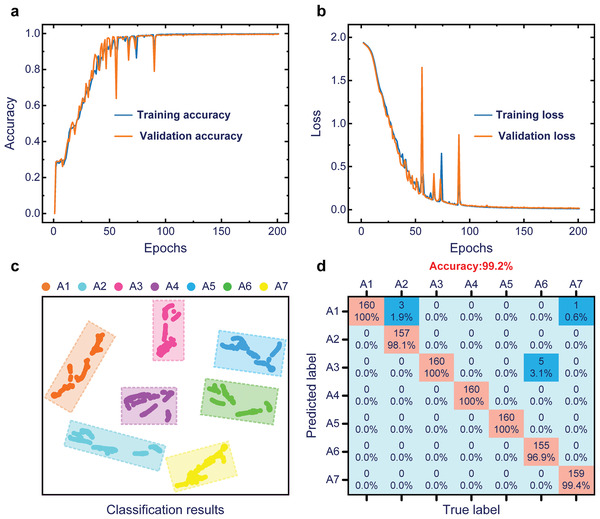
The identification result of the model. a) The accuracy of the model. b) The loss of the model. c) The classification results of the model. d) The confusion matrix of the model.

In CNN network, the parameters are set as follows: the layers of the convolutional layer, pooling layer, and fully connected layer are set to 2, 2, and 3, respectively; the Relu activation function is used; the optimizer is stochastic gradient descent; the batch_size is 128. In addition, to improve the diversity of data sets, the 5 set signals of the CSF‐TENG for each case were collected under the excitation frequency of 8 Hz and different excitation accelerations (0.4, 0.5, and 0.6 g). Thus, 11165 samples were obtained. According to the ratio of 7:2:1, the samples are divided into training sets, verification sets, and test sets (1120) to establish the defect detection model of the CSF‐TENG. As shown in **Figure** **5**a, when the epoch is 50, the accuracy of the model is about 80%, demonstrating that the model has excellent learning ability. As the epoch rises, the accuracy of the model gradually increases and tends to be stable. Due to the highly sensitive of TENGs and the effectiveness of the GAF and CNN, the accuracy of the model achieved 99.2%. As depicted in Figure 5b, the loss of the model decreases as the epoch increases and eventually stabilizes at around 0. To show the performance of the model more clearly, the classification results and confusion matrix of the model are illustrated in Figure 5c,d.

### 2.4 The Structure‐Crack Detection Digital Twin System Demonstration

A healthy energy harvesting device plays a vital role in the normal operation of sensor networks because the replacement frequency of power sources can be reduced. The long‐term operation or untimely maintenance of energy harvesting devices will weaken stability and reliability. Further, adopting the post‐fault maintenance method, equipment failures cannot be accurately predicted, resulting in a long maintenance cycle and high cost. Accordingly, the intelligent maintenance of energy harvesting devices is necessary. By constructing a twin of physical equipment and utilizing data interaction technologies, digital twin technology can realize the symbiosis of physical space and digital space, so corresponding intelligent services in the whole life cycle of physical equipment (design, manufacturing, operation, maintenance, and recovery) can be provided. Here, based on triboelectric sensors, GAF, CNN, and digital twin technology, the defect identification digital twin system of the CSF‐TENG was built.

**Figure 6:**
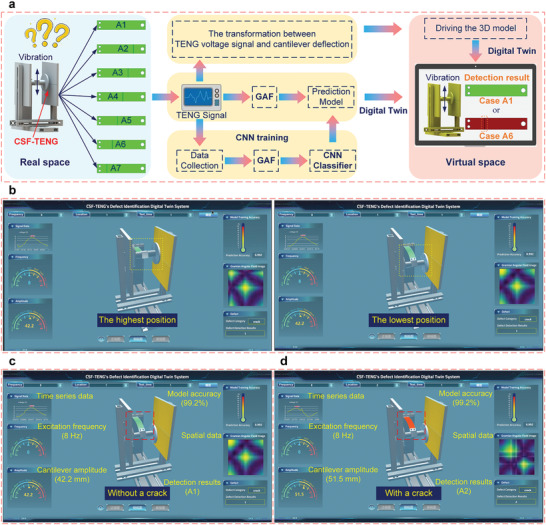
The defect identification digital twin system of the CSF‐TENG. a) The process from triboelectric sensory information collection to defect detection model training, crack identification, and 3D model driving in the digital twin system. To train the defect detection model based on convolutional neural network, the Gramian angular field is used to extract data features. b) Driving 3D model under Case A1. Displaying the crack recognition results c) under Case A1 and d) under Case A2.

The overall architecture of the defect identification digital twin system is divided into three layers (as illustrated in Figure S8, Supporting Information), that is, hardware layer, data layer, and application layer. Further, the function module mainly includes data management module, defect identification module, and 3D visualization module (as shown in Figure S9, Supporting Information). The data management module includes data acquisition, data storage, and data processing to achieve data transmission. The electrical signals of the CSF‐TENG are converted into image data by GAF, and then the defect types of cantilever beams are obtained through CNN. The 3D visualization module includes scene modeling, virtual and real synchronous mapping, and user interface design, which is utilized to realize the condition monitoring of the CSF‐TENG and display the defect identification results visually. The process of establishing the defect identification digital twin system of the CSF‐TENG is depicted in **Figure** **6**a.

In addition, it is worth mentioning that the electrical signals of the CSF‐TENG can not only be used to identify the defects of cantilevers, but also can be utilized to directly drive the twin model, realizing the synchronous operation of the CSF‐TENG in virtual space and real space. As described in Figure S10, Supporting Information, the relation between the output voltage of the CSF‐TENG and the cantilever's amplitude (end position) is obtained. Next, the fundamental mode will be dominant in response when the tip mass is greater than the mass of cantilevers,^[^ ^30^ ^]^ so the deflection can be expressed as:

(3) $$H\left(x,t\right)=q\psi \left(x\right)$$

where *q* is the modal coordinate, and *ψ*(*x*) is the first mode shape.

(4) $$\psi \left(x\right)=1-\cos\left(\frac{\pi x}{2L}\right)$$

Thus, the deflection of the cantilever at different positions is expressed as:

(5) $$H\left(x,t\right)=q\psi \left(x\right)=q\left[1-\cos\left(\frac{\pi x}{2L}\right)\right]$$

where the definitions of *L* and *x* in Equation (5) are shown in Figure S11, Supporting Information.

By Equation S9, Supporting Information, and Equation (5), the relation between the output voltage of the CSF‐TENG and the deflection of the cantilever was established, so the 3D model of the CSF‐TENG in the digital space can be driven directly by voltage signals (as shown in Figure 6b and Movies S1 and S2, Supporting Information), realizing the synchronous operation in virtual space and real space. Based on GAF, CNN, and the relation between the output voltages and the deflection, the defect identification digital twin system of the CSF‐TENG was created, and the system development environment is shown in Note S3, Supporting Information. As illustrated in Figure 6c, when the cantilever is crack‐free, its body appears green, and the identification result is displayed in the lower right corner of the system interface. Similarly, under Case A2, the cantilever is marked as red, and the detection results are exhibited in Figure 6d. Further, the system interface also displays triboelectric signal waveforms, frequency, amplitude, identification accuracy, and spatial data.

## 3 Conclusions

In summary, the defect identification digital twin system of the CSF‐TENG is proposed and investigated in this paper. The CSF‐TENG can be used to scavenge ambient energy for sensor networks or transmit sensory information. First, with and without a crack in cantilevers, the simulations were carried out. According to simulation results, the maximum change ratios of frequency and amplitude are 1.1% and 2.2%, respectively, causing obstacles in detecting defects of cantilevers via these variations. Accordingly, the method based on GAF and CNN was presented to establish the defect detection model. The 1D time series signal data of the CSF‐TENG is first converted into 2D image data by GAF, and then they are transmitted to the CNN for training to obtain the defect detection model with an accuracy of 99.2%. Further, the relation between the deflection of cantilevers and the output voltage of the CSF‐TENG was built, so the 3D model of the CSF‐TENG in the digital space can be driven directly by voltage signals. Based on GAF, CNN, and the relation between the output voltage and the deflection, the defect identification digital twin system of the CSF‐TENG was successfully created, which can show the defect recognition results and duplicate the operation of the CSF‐TENG in a real environment, realizing the intelligent maintenance of devices. In addition, the successful creation of the defect identification digital twin system of the CSF‐TENG can ensure the continuous service of sensor networks, which is of great significance to promote the development of smart cities, intelligent transportation, and so on.

## 4 Experimental Section

### Fabrication of the CSF‐TENG

The CSF‐TENG contained a fixed part and a moving part. For the moving part, all materials, such as sponge, PI, nickel cloth, and PTFE (as a negative triboelectric layer), were attached to the dielectric plate, which was connected to the frame and cantilever by screws. The sizes of the sponge, PI, nickel cloth, and PTFE are the same as the dielectric plate (the diameter is 40 mm). For the fixed part, all materials, such as PI, nickel cloth, and Gp (as a positive triboelectric layer), were attached to the electrode plate (the size is 80 × 100 mm), which contained upper and lower parts (the size is 80 × 49.8 mm). Thus, the size of Gp is 80 × 100 mm. But the size of PI and nickel cloth is 85 × 49.8 mm, which was designed to be wider to avoid the influence of electrode wires on the contact area of positive/negative triboelectric layers. In addition, the thicknesses of sponge, PI, nickel cloth, PTFE, and Gp are 2, 0.15, 0.1, 0.05, and 0.05 mm, respectively, and they were purchased from stores (as shown in Note S4, Supporting Information) and used without further treatment.

## Conflict of Interest

The authors declare no conflict of interest.

## Supporting information

Supporting Information

Supplemental Movie 1

Supplemental Movie 2

## Data Availability

The data that support the findings of this study are available from the corresponding author upon reasonable request.
