# Supplementary material for: Structure‐Crack Detection and Digital Twin Demonstration Based on Triboelectric Nanogenerator for Intelligent Maintenance
Source: Adv Sci (Weinh). 2023 Jul 6;10(26):2302443. doi: 10.1002/advs.202302443 (PMC10502813; doi:10.1002/advs.202302443)
Supplement: Supplementary file 1 — Supporting Information [file ADVS-10-2302443-s001.pdf]

ADVANCED SCIENCE
Open Access

Supporting Information

for *Adv. Sci.*, DOI 10.1002/advs.202302443

Structure-Crack Detection and Digital Twin Demonstration Based on Triboelectric Nanogenerator for Intelligent Maintenance

Chuanfu Xin, Zifeng Xu, Xie Xie, Hengyu Guo, Yan Peng, Zhongjie Li, Lilan Liu* and Shaorong Xie**

Structure-crack Detection and Digital Twin Demonstration based on Triboelectric Nanogenerator for Intelligent Maintenance

Chuanfu Xin, Zifeng Xu, Xie Xie, Hengyu Guo, Yan Peng, Zhongjie Li, Lilan Liu,* Shaorong Xie**

C. Xin, Z. Xu, X. Xie, Z. Li, L. Liu
School of Mechatronic Engineering and Automation, Shanghai University, Shanghai 200444, P.R. China
E-mail: lizhongjie@shu.edu.cn; lancy@shu.edu.cn

S. Xie
School of Computer Engineering and Science, Shanghai University, Shanghai 200444, P.R. China
E-mail: srxie@shu.edu.cn

Z. Li, Y. Peng
Institute of Artificial Intelligence, Shanghai University, Shanghai 200444, P.R. China

Z. Li
Engineering Research Center of Unmanned Intelligent Marine Equipment, Shanghai University, Shanghai 200444, P.R. China

H. Guo
Department of Applied Physics, Chongqing University, Chongqing 400044, P.R. China

Z. Xu, L. Liu
Shanghai Key Laboratory of Intelligent Manufacturing and Robotics, Shanghai University, Shanghai 200444, P.R. China

* Corresponding author

Supplementary Information

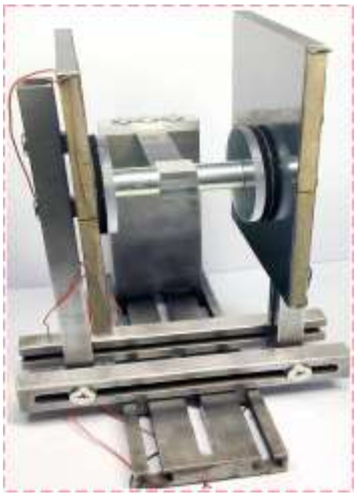

Figure. S1 The object mechanical model of the device.

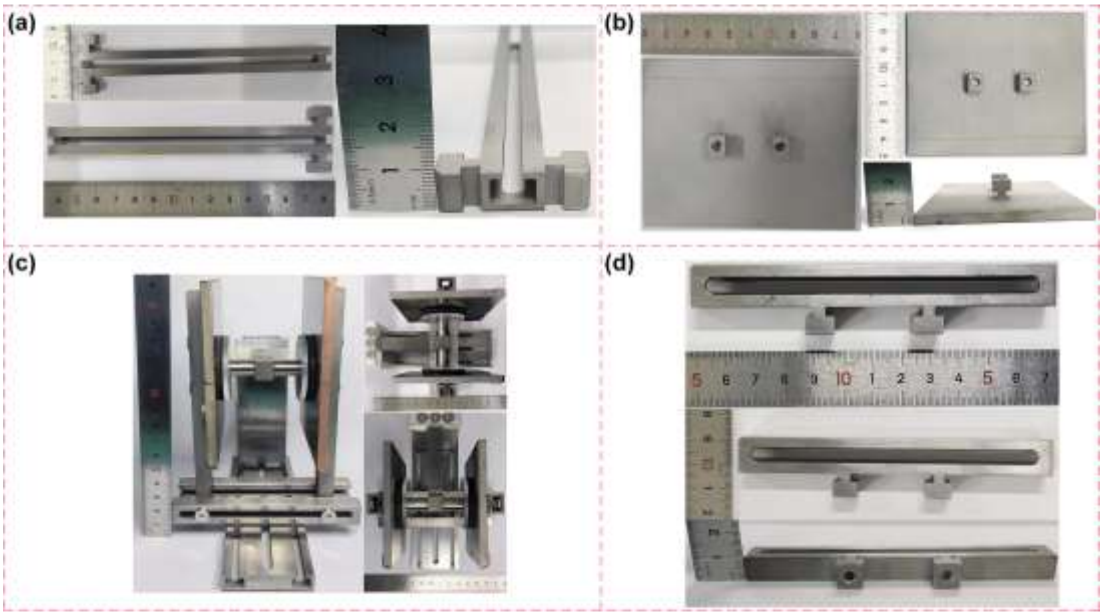

Figure. S2 The overall dimensions of the device and different boards. (a) the dimensions of slide rail-3; (b) the dimensions of the electrode plate; (c) the dimensions of the device; (d) the dimensions of slide rail-2.

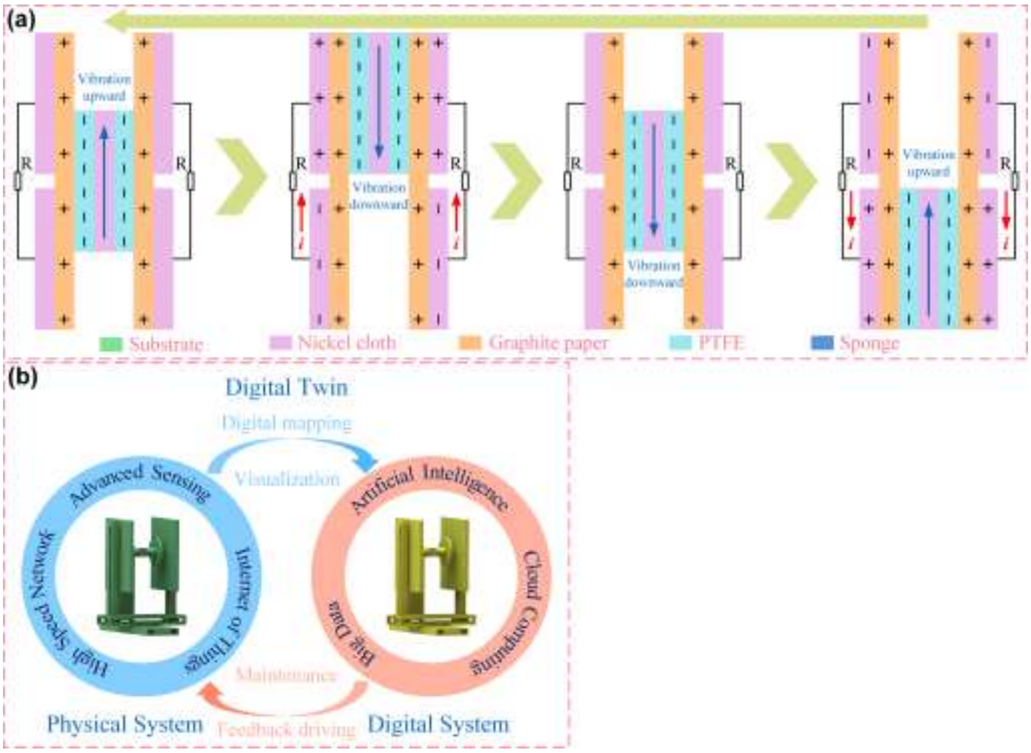

Figure. S3 The working principle of the CSF-TENG and architecture of digital twin. (a) the working principle of the CSF-TENG; (b) the architecture of digital twin.

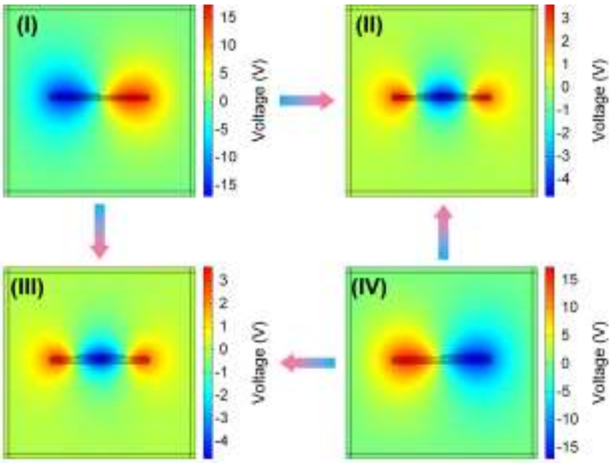

Figure. S4 The output voltage simulation of the CSF-TENG.

Under the triboelectric layers of graphite paper and PTFE, we performed simulations in COMSOL Multiphysics 5.6. First, the sizes of graphite paper, PTFE, and electrodes are set to 100×80 mm, a diameter of 40 mm, and 49.8×80 mm, and the distance between two electrodes is 0.4 mm. Then, the equally positive and negative charges are added to the graphite paper and PTFE, which remain constant in all simulations. Based on different positions of the PTFE triboelectric layer, the simulation results are shown in Supplementary Figure 4.

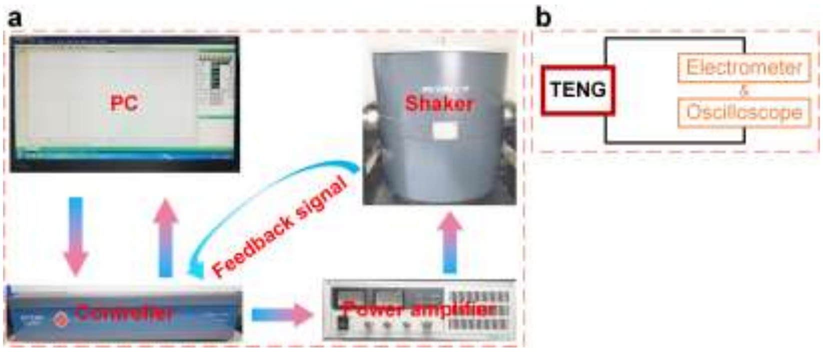

Figure. S5 The experimental platform and testing circuits. (a) the experimental platform of the CSF-TENG; (b) the testing circuits of the CSF-TENG.

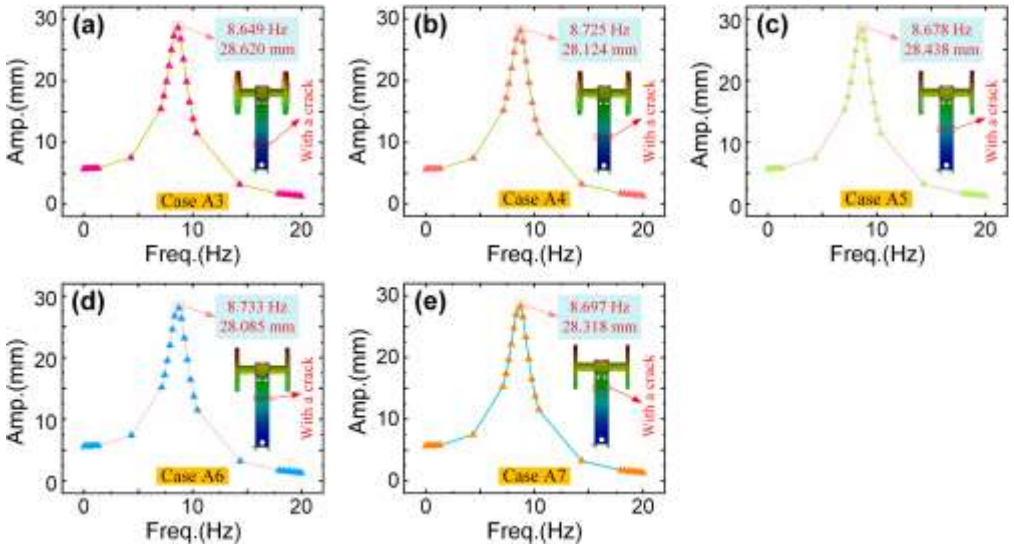

Figure. S6 The simulation results of cantilevers under (a) Case A3, (b) Case A4, (c) Case A5, (d) Case A6, and (e) Case A7.

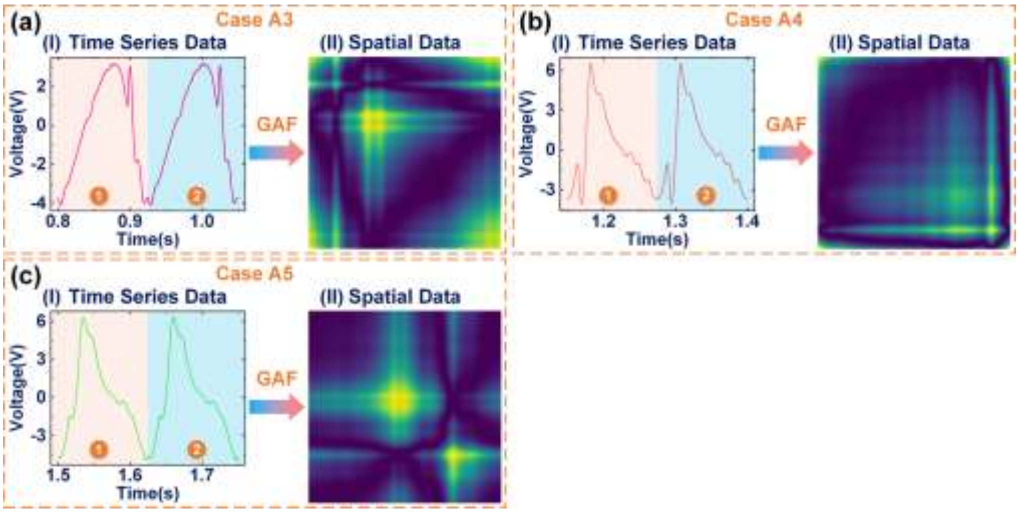

Figure. S7 The results of converting time series data into spatial data under (a) Case A3, (b) Case A4, and (c) Case A5.

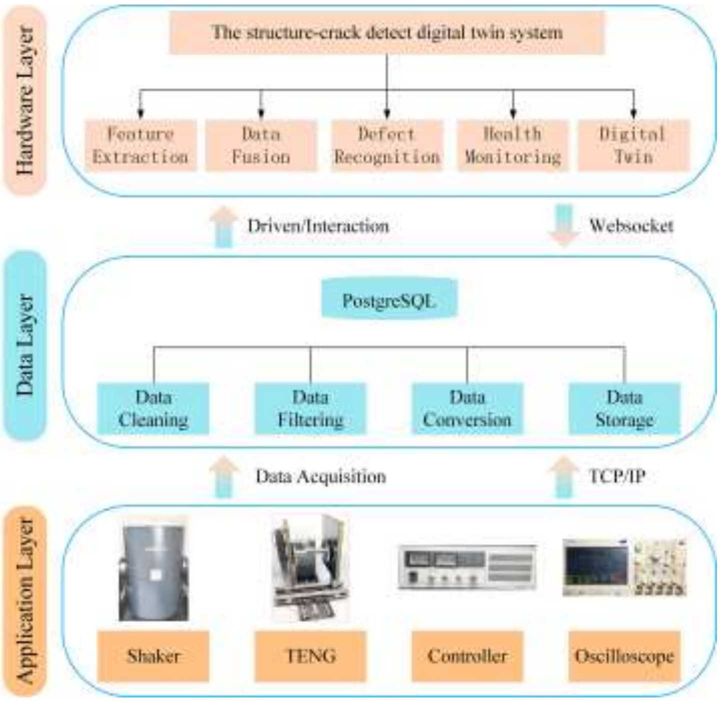

Figure. S8 The overall architecture of the defect identification digital twin system.

Digital twin technology describes the state of integration of information space and physical world. The overall architecture of the digital twin system proposed in this paper is divided into three layers, including (1) Hardware Layer, comprising the shaker, cantilevers with different cracks, data acquisition equipment, etc.; (2) Data Layer, providing data processing and storage services; (3) Application Layer,

consisting of data conversion, data visualization, cantilever defect identification, twin model verification, etc.

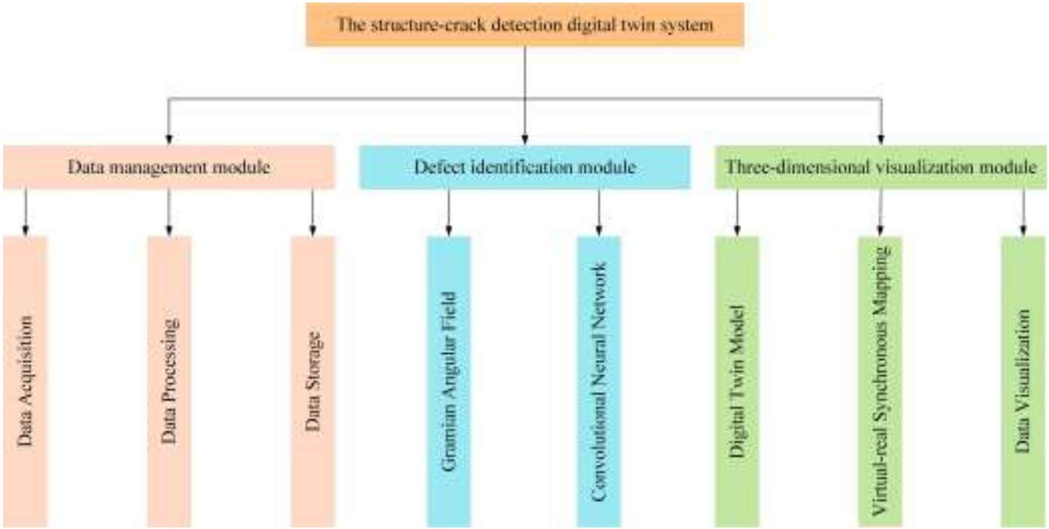

Figure. S9 The function modules of the structure-crack detection digital twin system.

The structure-crack detection digital twin system is divided into data management module, defect identification module, and three-dimensional visualization module. The data management module includes data acquisition, data storage, and data processing, which is used to drive the system operation. The defect recognition module is composed of gramian angular field (GAF) and convolutional neural network (CNN), which first utilizes GAF to convert the vibration timing sequence data of cantilevers into image data, and then uses CNN to identify defects. The 3D visualization module includes scene modeling, virtual and real synchronization mapping, and user interface design, etc., which is used to realize the condition monitoring of cantilevers and display the identification results of cantilevers.

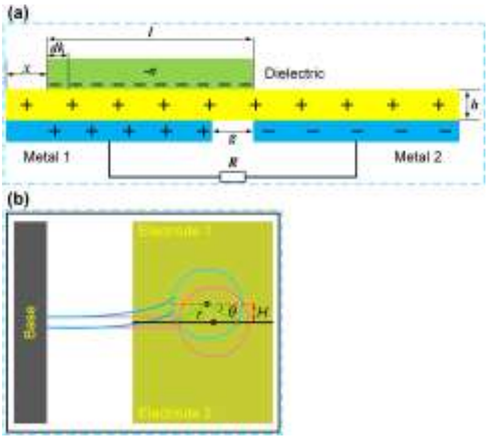

Figure. S10 In CSF-TENG, (a) the working principle; (b) the relation between the output voltage and amplitude.

As shown in **Figure. S10(a)**, we assume that dk is a small region in the bottom dielectric surface, which contains tribo-charges with a density of σ. Therefore, the total charges on metal 1 and metal 2 can be described as σwdk. In addition, the thickness of the positive triboelectric layer and electrode gap are h and g, respectively. Thus, the total charges of metal 1 and metal 2 (dQ_1 and dQ_2) can be calculated by using the following equations under the short-circuit conditions,

$$dQ_1 = \frac{\sigma wdk}{1+\frac{C_2(k)}{C_1(k)}} \tag{1}$$

$$dQ_2 = \frac{\sigma wdk}{1+\frac{C_1(k)}{C_2(k)}} \tag{2}$$

where $C_i(k)$ is the capacitance between this small region dk and metal i (i is 1, and 2.), and w is the width of triboelectric layers.

According to the principle of superposition of electrostatic fields, the total charges on metal 1 and metal 2 can be shown as:

$$Q_1 = \sigma w \int_0^l \frac{dk}{1+\frac{C_2(k)}{C_1(k)}} \tag{3}$$

$$Q_2 = \sigma w \int_0^l \frac{dk}{1+\frac{C_1(k)}{C_2(k)}} \tag{4}$$

where l is the length of the dielectric layer.

Therefore, the short-circuit charges (ΔQ) between metal 1 and metal 2 can be shown as:

$$\Delta Q = \sigma w \left(\int_0^l \frac{dk}{1+\left(\frac{C_2(k)}{C_1(k)}\right)_{L=x}} - \int_0^l \frac{dk}{1+\left(\frac{C_1(k)}{C_2(k)}\right)_{L=0}} \right) \tag{5}$$

When the cantilever is excited, the dielectric plate swings in the vertical direction, so the ambient energy is converted into electrical energy. According to the working

principle of free-standing TENGs, the relation between peak-to-peak voltage (V) and the amplitude (H) of the dielectric plate can be explained with the following formula:

$$V = \frac{\Delta Q}{C} = \frac{\sigma \Delta S}{C} \tag{6}$$

where C is the equivalent capacitance between the two electrodes, and ΔS is calculated by the following formula:

$$\Delta S = \frac{\pi \theta r^2}{180^\circ} + Hr\cos(\theta) \tag{7}$$

In addition, θ can be obtained with the following equation:

$$\sin(\theta) = \frac{H}{r} \tag{8}$$

As shown in **Figure. S10(b)**, r and H are the radius and amplitude of the dielectric plate.

The final expressions of the peak-to-peak voltage (V) and amplitude (H) are expressed in Equation (9).

$$V = \frac{\sigma}{C}\left(\frac{\pi r^2 \arcsin(H/r)}{180} + Hr\cos\left(\arcsin\left(H/r\right)\right)\right) \tag{9}$$

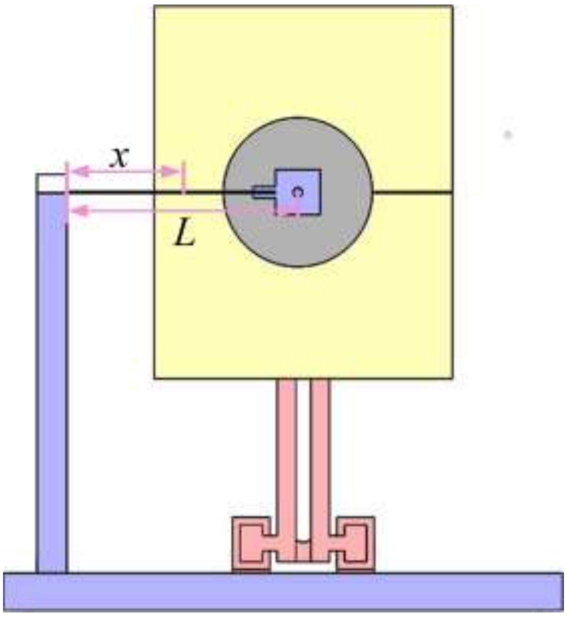

Figure. S11 The definitions of L and x.

Supplementary Note 1

(i) Normalization of one-dimensional time series signal data

$$x_{-1} = \frac{(x_i - \max(X)) + (x_i - \min(X))}{\max(X) - \min(X)}, i = 1, 2, 3, \ldots, n \tag{10}$$

$$x_0 = \frac{x_i - \min(X)}{\max(X) - \min(X)}, i = 1, 2, 3, \ldots, n \tag{11}$$

where X (X = {x_1, x_2, x_3, ..., x_n}) is the time series signal data, x_i is the i-th data of a time series signal, and x is normalized sequence data.

(ii) Encoding normalized sequence data *X* in polar coordinate

The reacquired voltage value is encoded as angle cosine, and the corresponding time is encoded as radius.

$$\begin{cases} \phi_i = \arccos(x_i), -1 \le x_i \le 1, x_i \in X \\ r_i = \dfrac{t_i}{N}, t_i \in N \end{cases} \tag{12}$$

where N is the normalization factor, and t_i is the time point corresponding to x_i.

(iii) The definition of gramian angular field

Gramian angular field is defined as using trigonometric sum or difference between each point to identify the correlation, which is divided into gramian angular summation fields (GASF) and gramian angular difference fields (GADF). The definition equations of GASF and GADF are as follows:

$$GASF = \begin{Bmatrix} \cos(\phi_1 + \phi_2) & \ldots & \cos(\phi_1 + \phi_n) \\ \cos(\phi_2 + \phi_1) & \ldots & \cos(\phi_2 + \phi_n) \\ \ldots & \ldots & \ldots \\ \cos(\phi_1 + \phi_2) & \ldots & \cos(\phi_n + \phi_n) \end{Bmatrix} \tag{13}$$

$$GADF = \begin{Bmatrix} \cos(\phi_1 - \phi_2) & \ldots & \cos(\phi_1 - \phi_n) \\ \cos(\phi_2 - \phi_1) & \ldots & \cos(\phi_2 - \phi_n) \\ \ldots & \ldots & \ldots \\ \cos(\phi_1 - \phi_2) & \ldots & \cos(\phi_n - \phi_n) \end{Bmatrix} \tag{14}$$

According to the above equations, the operation of GAF adopts the gram matrix, namely, the diagonal of the matrix is formed by the original value of the scaled time series.

$$\begin{cases} diag(GASF) = \{\cos(2\phi_1), \ldots, \cos(2\phi_n)\} \\ diag(GADF) = 0 \end{cases} \tag{15}$$

Gram matrix, which can reflect the relation between vectors, is composed of the inner product of two groups of vectors. Thus, the matrix is known as the gram matrix of the *k* vectors, which is composed of the inner product of any *k* vectors in the n-dimensional Euclidean space.

$$\Delta(\alpha_1,\alpha_2,\ldots,\alpha_k)=\begin{Bmatrix}(\alpha_1,\alpha_1) & \cdots & (\alpha_1,\alpha_k)\\ \vdots & \ddots & \vdots\\ (\alpha_k,\alpha_1) & \cdots & (\alpha_k,\alpha_k)\end{Bmatrix} \tag{16}$$

Supplementary Note 2

(i) Convolutional layer

Convolution layer, which is used to extract image features, is composed of convolution kernels and activation functions. The calculation in the convolution layer is to convolve feature vectors with convolution kernels, and results are transformed through activation functions to obtain new features.

$$y_a^l = f(\omega_a^l \otimes x_a^l + b_a^l) \tag{17}$$

where y_a^l is the output of the *a*-th neuron in *l* layer, ω_a^l is the weight of the convolution kernel, x_a^l is the input, b_a^l is the bias, $\otimes$ is a convolution operation, and *f* (•) is the activation function.

(ii) Pooling layer

The output will enter the pooling layer after the feature map vector is convoluted in the convolutional layer. Pooling operations can make the feature map smaller, diminish the amount of data, and decline the computational complexity of CNN. Simultaneously, it can compress image features, retain effective information, reduce calculation time, and accelerate calculation speed.

$$x_i^l = \beta_i^l down(x_i^{l-1}) + b_i^l \tag{18}$$

where *down*(•) is the down-sampled function, x_i^{l-1} is the *i*-th feature map in *l*-1 layer, β_i^l is the magnification factor of the *i*-th feature map in *l* layer, b_i^l is the bias coefficient of the *i*-th feature map in *l* layer, and x_i^l the *i*-th feature map in *l* layer.

(iii) Fully connected layer

Fully connected layer is to connect all neurons in the front and back layers, scale the input features into one-dimensional vectors, and then classify them with the softmax function to obtain final classification results.

$$x_i^l = f\left(\sum_{j=1}^{n} x_j^{l-1} \omega_{i,j}^{l-1} + b_i^l\right) \tag{19}$$

where n is the number of neurons in l-1 layer, x_j^{l-1} is the output of the j-th neuron in l-1 layer, $\omega_{i,j}^{l-1}$ is the connection weight between the j-th neuron in l-1 layer and the i-th neuron in l layer, b_i^l is the bias of the i-th neuron in l layer, and x_i^l is the output of the i-th neuron in l layer after passing through fully connected layer.

(iv) Activation function

The convolutional layer, pooling layer, and fully connected layer are all linear operations in CNN, and the simple stacking of linear operations cannot solve complex nonlinear classification problems. However, the activation function (such as Sigmoid, Tanh, Relu, and Leaky Relu) can realize the nonlinear transformation in the process of data transmission, so that CNN can learn more complex features.

Supplementary Note 3

Server running environment: CPU: 12th Gen Intel(R) Core (TM) i9-12900H 2.50 GHz; Display Card: Nvidia GeForce GTX1060; Memory: 16 GB; Hard Disk: 4 TB; Operating System: Windows 10; Data Base: PostgreSQL 11.5; Software Development Environment: Pycharm 2022.2; Python Interpreter Version: 3.8.2; Tensorflow 2.7.1.

Supplementary Note 4

PI was purchased from Guangzhou (China) Beilong Electronics Co., Ltd. Sponge was purchased from Pinrao environmental protection material store (Taobao). PTFE was purchased from 3j flagship store (Taobao). Graphite paper was purchased from Guangsheng Jiajin Metal New Material Store (Taobao).
